# Book Reviews

**Published:** 1896-01

**Authors:** 


A Hand-Book of Medical Diagnosis. By James B. Herrick, M. D., Adjunct Professor of Medicine, Rush Medical College, Chicago. In one 12mo volume of 429 pages, with eighty engravings and two colored plates. Cloth, $2.50. Philadelphia: Lea Brothers & Co., Publishers. 1895.

Artfulness in diagnosis is an essential to therapeutic success, and it is well to have all the methods of the former discussed from various standpoints. Herrick is a new contestant for professional favor in this field, but he greets his audience with becoming modesty and presents his observations with a clearness and conciseness which demonstrate that he is a master of his subject.

His book is intended for students, hence is to be judged from a viewpoint altogether different from that of the larger and completer treatises that are written for more advanced readers. The author has aimed to group into compact form the principal facts that aid in diagnosis; that is, those to be derived from clinical observation, from chemistry, bacteriology and the microscope. He expresses a hope that the volume will be more than a quiz-compend and that it will stimulate to further study in larger works.

Taking Herrick's observations on diseases of the heart and pericardium as an example, we should say that he had reasonably succeeded in his expectations and desires. He describes the physical examination of the heart in an admirable manner, without a superfluous sentence, and no student can fail to derive valuable information from its study.

We commend the work especially to undergraduates, but may add that the busy physician will find it an excellent prompter to his memory.

---

Physical and Natural Therapeutics. The Remedial Use of Heat, Electricity, Modifications of Atmospheric Pressure, Climates and Mineral Waters. By Georges Hayem, M. D., Professor of Clinical Medicine in the Faculty of Medicine of Paris. Edited with the assent of the author, by Hobart Amory Hare, M. D., Professor of Therapeutics in the Jefferson Medical College of Philadelphia. In one handsome octavo volume of 414 pages, with 113 engravings. Cloth, $3.00. Philadelphia: Lea Brothers & Co., Publishers. 1895.

The above title gives a synopsis of the scope of this interesting work which, it will be observed, is different from the ordinary therapeutical treatise. It is devoted to the consideration of remedies outside of the materia medica in their applicability to the treatment of disease. Every physician understands that there are many remedies more potent than mere drugs. Fresh air, exercise, pure water, a light heart and a clear conscience are all good medicines.

This work, written by an able French therapeutist and edited by an equally distinguished American author in the same department of medicine, it cannot fail to find favor in the United States.

It is divided into six parts—namely, part I., atmospheric pressure as a therapeutic agent; part II., climate; part III., thermic agents; part IV., hydro-therapeutic measures; part V., mineral waters, and part VI., electricity.

The section on climate has been rewritten by the editor and now for the first time places the abundant resources of our own country, in this particular, at the intelligent command of the American profession. Medical electricity is very properly considered as the closing section, being of least consequence, although the space devoted to it is out of proportion to its relative importance.

This is the kind of a book that every physician, no matter what his specialty, needs to have at ready command for reference and consultation.

---

Modern Materia Medica with Therapeutic Notes. For the use of Practitioners and Students of Medicine. By Dr. Otto Roth. Seventh edition. Revised by Dr. Gregor Smith, Würzburg. One volume of 467 pages, octavo, muslin binding. Price, $2.00. New York: William Wood & Co. 1895.

This book was translated some years ago for Wood's medical and surgical monographs. It was soon afterward reprinted in book form and a translation of the seventh edition is now offered to the profession. The arrangement of its contents is as follows: I. The various remedial agents grouped according to their physiological and therapeutic action. II. Drugs and other remedies, alphabetically arranged, with remarks on their physiological action, therapeutic use and dosage. III. Remedies most commonly used for subcutaneous injection. IV. The most commonly employed remedies for inhalation. V. Therapeutic notes. VI. Table of maximum doses for an adult. VII. Dosage for children of various drugs. VIII. Therapeutic index.

The grouping of the subjects in this book is excellent, many of the prescriptions published are useful and altogether it is a book that will prove of great service to the practiser of general medicine.

---

TEXT-BOOK OF PHYSIOLOGY. By MICHAEL FOSTER, M. D., F. R. S., Prelector in Physiology and Fellow of Trinity College, Cambridge, Eng. New (sixth) American edition with notes and additions. In one octavo volume of 922 pages, with 257 illustrations. Cloth, $4.50; leather, $5.50. Philadelphia: Lea Brothers & Co., Publishers. 1895.

This new sixth edition of this work will be most welcome to every teacher and student of physiology. The author enjoys the distinction of being the leading English physiologist, hence his book is the leading text-book in use by English-speaking students abroad. In this edition it is asserted that every page has been subjected to careful scrutiny, useless verbiage omitted, obscure sentences revised or rewritten, typographical errors corrected, histological details abridged, theoretical parts expunged and many alterations and additions made, bringing the book forward to the present time. It is adapted to the use of junior as well as advanced students and it is one of the best single volume treatises for the practiser of general medicine. Many of the illustrations have been reëngraved, while the general style and price of the book remain as before. Foster is especially interesting in dealing with the special senses, but we think the space he devotes to the physiology of reproduction is rather too scanty, considering the importance of the subject. In an appendix, however, he treats fully the chemical basis of the animal body, which is an exceedingly interesting section.

---

TRANSACTIONS OF THE ASSOCIATION OF AMERICAN PHYSICIANS. Tenth session, held at Washington, D. C., May 30 and 31, 1895. Philadelphia: Wm. J. Dornan, Printer. 1895.

This volume of the transactions of this eminent society is an interesting record of its work during its last annual meeting. The papers read cover a wide range of subjects, and many of them were ably discussed. A paper, entitled Two cases of fat necrosis, by Drs. Charles G. Stockton and Herbert U. Williams, of Buffalo, possesses much interest and is well illustrated. Another paper that invites the study of the curious, and is also not without its practical side, is that by Dr. Charles Cary, of Buffalo, on The cause of the disparity found, both in health and disease, on physical examination of the upper portion of the chest. This paper contains two beautiful illustrations, showing casts of the bronchial tubes of the right and left lungs.

The volume is uniform in style and type with those that have preceded it.

---

A Text-Book of Practical Medicine. Designed for the use of Students and Practitioners of Medicine. By Alfred L. Loomis, M. D., LL. D., Professor of Pathology and Practical Medicine in the Medical Department of the University of the City of New York ; Visiting Physician to Bellevue Hospital, etc. Revised and enlarged, with 207 illustrations and one chromo-lithographic plate. Eleventh edition, 1134 pages. Price, cloth, $6.00 ; leather, $7. New York: William Wood & Co. 1895.

The first edition of this treatise appeared in 1884, since which time ten others have been put forth—an average of one edition a year. This is evidence of the popularity of the work. Dr. Loomis was engaged in the revision of the eleventh edition at the time of his fatal illness and had nearly completed it. Since his death only such alterations and additions have been made as seemed necessary.

Dr. Loomis was a teacher of forceful and persuasive powers, and he has carried his personality into this work, which has steadily maintained its hold as a text-book in the schools in spite of the active competition of many other worthy treatises. To those who have possessed the earlier editions this revision will become almost a necessity, while those who are strangers to the work will find it full of interest and teeming with practical teachings.

